# Exclusion of older adults from clinical trials in cancer-related pain

**DOI:** 10.3389/fmed.2022.945481

**Published:** 2022-08-04

**Authors:** Krzysztof Krysa, Ewa Kowalczyk, Jan Borysowski, Mieszko Lachota, Tomasz Pasierski

**Affiliations:** ^1^First Military Hospital, Lublin, Poland; ^2^Clinical Research Development Centre, Medical Research Agency, Warsaw, Poland; ^3^Department of Clinical Immunology, Medical University of Warsaw, Warsaw, Poland; ^4^Centre for Studies on Research Integrity, Institute of Law Studies, Polish Academy of Sciences, Warsaw, Poland; ^5^Department of Medical Ethics and Palliative Medicine, Medical University of Warsaw, Warsaw, Poland

**Keywords:** elderly, older adults, clinical trial, cancer pain, cancer-related pain, enrollment criteria

## Abstract

Pain is one of the most common symptoms in cancer patients including older adults. The objective of this study was to evaluate the enrollment criteria that can limit the inclusion of older adults in clinical trials concerning cancer-related pain (CRP). The study included 356 trials registered with ClinicalTrials.gov. Our primary outcome measures were the proportion of trials that excluded patients based on upper age limits (80 years of age or less), strict organ-specific exclusion criteria, broad and imprecise criteria, and inadequate performance score. One hundred and twenty-six trials (35.4%) had upper age limits. Strict exclusion criteria were used in 95 (26.7%) trials. Broad and imprecise exclusion criteria were listed in 57 (16.2%) trials. Low performance score was used as an exclusion criterion in 4 trials (1.1%). Overall, in 241 trials (67.7%) there was either an upper age limit or at least one strict or broad and imprecise exclusion criterion, or a criterion involving the performance status. The odds of excluding older adults were significantly higher in certain neoplasm types, study objectives, intervention types, and center locations. In conclusion, considerable proportion of recent clinical trials concerning CRP either explicitly exclude older adults or create high risk of such exclusion which substantially limits the evidence base for the treatment of such patients in clinical practice. Sponsors and investigators should consider careful modification of the enrollment criteria to improve the inclusion of older individuals who make up the major proportion of cancer patients population.

## Introduction

Pain is one of the most common symptoms in cancer patients (1). It frequently occurs both in patients with solid tumors (2) and those with hematological malignancies (3). A recent meta-analysis showed that the prevalence of pain in patients receiving anticancer treatment, after the treatment, and those with advanced, metastatic, or terminal disease is 55, 39.3, and 66.4%, respectively (4). Pain is one of the most significant factors reducing the quality of life of cancer patients (5).

Treatment of cancer-related pain (CRP) is a major clinical challenge; a recent systematic review showed that ~30% of cancer patients do not receive analgesic treatment adequate to the pain intensity (6). Inadequate control of CRP can have a number of serious consequences including substantial disturbance of patient daily activities (7), reduced compliance with anticancer treatments (8), higher medical costs (9), and higher level of depression and anxiety of family caregivers of patients (10).

Pain is even the greater clinical problem in older adults with cancer (11–13). In view of a number of factors such as renal or hepatic impairment, other co-morbidities, and polypharmacy, older cancer patients can respond to various treatments differently to younger ones (14). Therefore, to ensure optimal care of older patients, doctors need the data on the benefits and harms of analgesic treatments coming from clinical trials involving such individuals. However, it is known that older patients have been underrepresented in clinical trials concerning cancer (15). One of the main barriers which can limit the enrollment of older cancer patients in clinical studies are stringent eligibility criteria (16–20).

However, to our knowledge, no studies have yet been performed to evaluate the enrollment criteria in clinical trials concerning CRP. We hypothesized that in many clinical trials concerning CRP these criteria also can limit the enrollment of older individuals. To verify our hypothesis, we assessed the enrollment criteria in clinical trials related to CRP that have been registered with ClinicalTrials.gov (CT.gov), the most comprehensive register of clinical studies in the world (https://www.clinicaltrials.gov/). We examined both the age limits used in the trials and the criteria that may indirectly limit the inclusion of older adults.

## Methods

### Selection of clinical trials

Pain in cancer patients can have a wide range of causes. In this study we classified as CRP both pain caused by the neoplasm itself (including the metastases) and that resulting from anticancer treatment including pharmacotherapy, radiotherapy, and surgery. However, it is estimated that ~9% of cancer patients experience pain that is unrelated to either the cancer itself or anticancer treatment (21). Therefore, to select eligible trials, in each case we checked whether a trial record contains information that a given study concerns CRP, and not pain due to other etiologies (e.g., pain due to osteoarthritis).

Clinical trials concerning CRP were searched for in CT.gov. In order to identify eligible trials we used the search term “Cancer pain” (field “Condition or disease”) which results in the selection of trials not only based on this specific term, but also its synonyms used by the CT.gov search engine including “Cancer related pain,” “Tumor related pain,” “Oncological pains,” “Oncology pains,” “Cancer associated pain,” and “Neoplasm related pain'. We used the following inclusion criteria: (1) Interventional studies; (2) Study start date on 01/01/2014 or later; (3) Primary purpose “Prevention,” “Treatment,” or “Supportive Care”. We excluded studies with the recruitment status “Suspended” or “Withdrawn”, trials performed on healthy volunteers, trials concerning pain other than CRP as defined above and pediatric clinical trials (however, if a trial enabled the enrollment of both adolescents and adults, it was included).

### Data extraction and analysis

From record of each eligible study we extracted the following data: CT.gov identifier, recruitment status, sponsor(s), cancer type, intervention type, phase, enrollment, allocation, primary purpose, study start date. We also extracted the data on basic pain characteristics and relevant enrollment criteria, especially the age limits and the criteria related to bone marrow, liver, kidney, the cardiovascular system, and the pulmonary system. Moreover, we recorded psychiatric diseases and prior or concurrent malignancies listed as the exclusion criteria.

In the analysis of the enrollment criteria we used a classification system developed by Lewis et al. (22) and used in other studies on the exclusion of the elderly from clinical trials (19). In brief, the exclusion criteria related to any of the above-mentioned organs, systems, and diseases have been divided into two main categories—moderate and strict. Strict criteria required normal or nearly normal organ functions and/or laboratory parameters, while moderate criteria permitted the inclusion of patients with mild abnormalities. If a trial had both moderate and strict exclusion criteria related to the same organ or system, it was classified as having strict criteria. Of note, the classification includes “Mental illness making informed consent impossible” as a criterion related to psychiatry. Therefore, we considered cognitive impairment as one of such criteria (the classification does not include a separate category related to neurological diseases). Full list of the moderate and strict exclusion criteria related to different organs and systems is available at (https://theoncologist.onlinelibrary.wiley.com/doi/suppl/10.1634/theoncologist.2014-0093).

Apart from the exclusion criteria concerning function of specific organs and systems listed in the above-mentioned classification, we also recorded broad and imprecise criteria open to investigators' interpretation (e.g., “the presence of any significant disease”). Moreover, we extracted the data about the patient performance score listed as an exclusion criterion. When analyzing the data on the performance score, we assumed that grade 0 in the Eastern Cooperative Oncology Group (ECOG) scale corresponds to score 100 in Karnofsky scale (KS), ECOG grade 1 is equal to score 80–90 in KS, ECOG grade 2 corresponds to score 60–70 in KS, ECOG grade 3 is equal to score 40–50 in KS, and ECOG grade 4 corresponds to score ≤30 in KS (19).

Our primary outcome measures included the proportion of trials that excluded patients based on strict organ/system-specific exclusion criteria, broad and imprecise criteria, inadequate performance score (grade 2 or more in the ECOG scale), and arbitrary upper age limits (80 years of age or less).

### Statistical analysis

Discrete variables were presented as absolute numbers and percentages, whereas continuous variables as medians with interquartile ranges. Statistical calculations were performed using R package (23). “Tidyverse” was used to process all the data (24). Multivariate logistic regression was employed to determine the relationship between the primary outcome measures and other characteristics of clinical trials. Temporal trends regarding the primary outcome measures were evaluated using univariate logistic regression. “Jtools” R package was used to export the created models to.xlsx format (25). Chi-Square test was employed to assess whether the presence of strict or broad exclusion criteria depended on the presence of pre-defined age limits of 80 years old or lower. In all analyses, *p* < 0.05 was considered a statistically significant threshold.

## Results

### Characteristics of the included clinical trials

**Figure 1:**
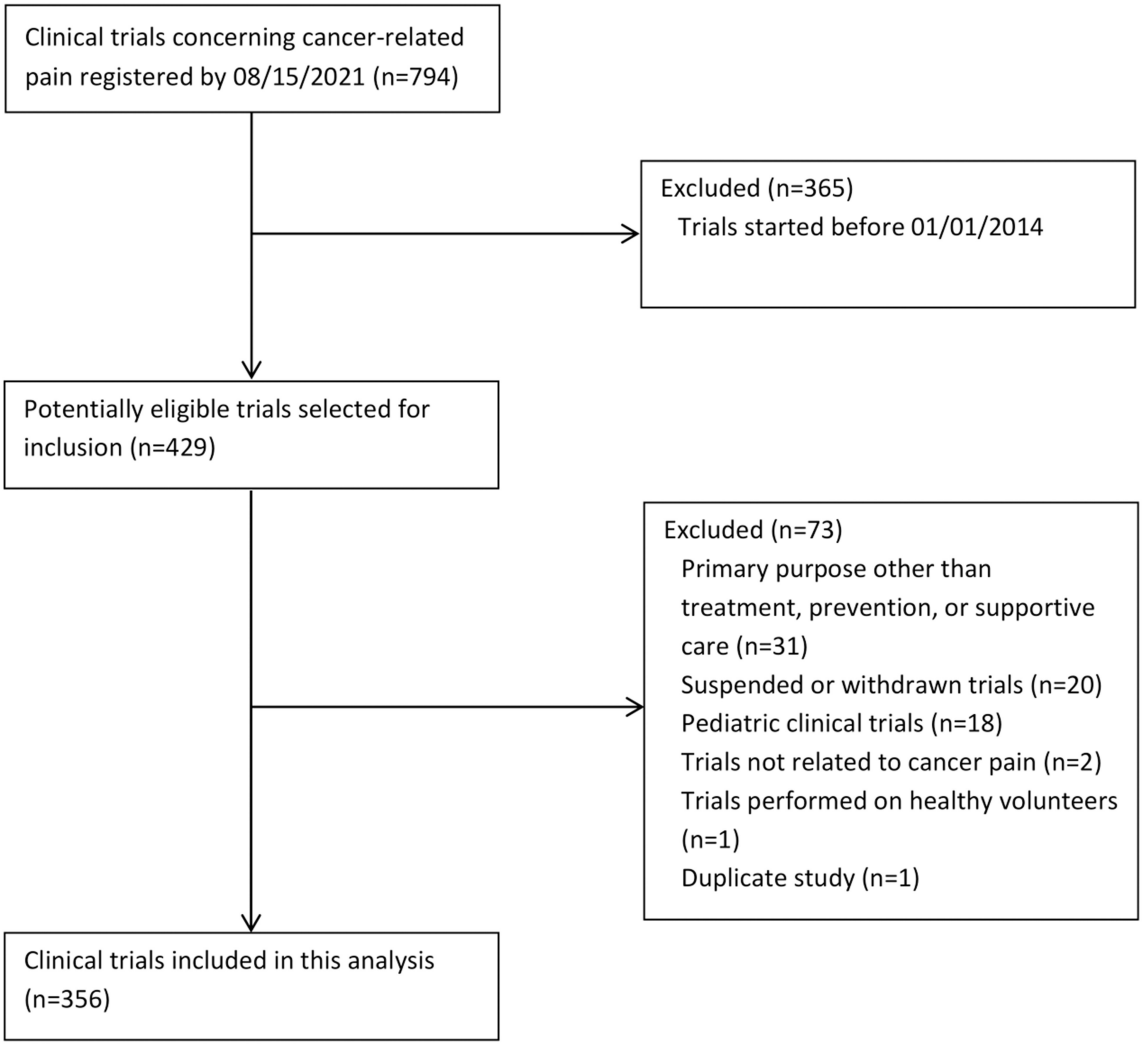
Selection of eligible clinical trials concerning cancer-related pain.

Clinical trials of interventions being evaluated in the prevention, treatment, or supportive care of patients with CRP were searched for in CT.gov (search date 08/15/2021). The selection of eligible trials is shown in Figure 1. Our initial search yielded 794 trials. Eventually we included 356 trials.

The characteristics of the included trials are shown in Table 1. Therapeutic intervention that was used most often were drugs (*n* = 134; 37.6%). Many trials enabled the enrollment of patients with any neoplasm type (*n* = 122; 34.3%). When a specific neoplasm type or neoplasm location was listed as the targeted condition, it was most often breast cancer (*n* = 104; 29.2%) followed by gastrointestinal cancer (*n* = 44; 12.4%), and head and neck cancer (HNC; *n* = 26; 7.3%). The median number of participants was 68 (IR range 37.5–120). The most common center locations included the USA (*n* = 111; 31.2%), Egypt (*n* = 38; 10.7%), and China (*n* = 36; 10.1%). Most of the trials were entirely funded by non-industrial sources (*n* = 320; 89.9%).

**Table 1:** Characteristics of clinical trials in cancer-related pain.

	***n***	**%**
**Sponsor**		
Industry^a^	36	10.1
Non-industry	320	89.9
**Phase**		
1	9	2.5
1/2	9	2.5
2	38	10.7
2/3	11	3.1
3	33	9.3
4	31	8.7
Not applicable	225	63.2
**Status**		
Completed	145	40.7
Recruiting	93	26.1
Unknown	44	12.4
Not yet recruiting	37	10.4
Terminated	19	5.3
Active, non-recruiting	17	4.8
Enrolling by invitation	1	0.3
**Center location**		
North America	132	37.1
Europe	79	22.2
Asia/Far East	69	19.4
Middle East/North Africa	60	16.9
Other	9	2.5
International trials	5	1.4
Unknown	2	0.6
**Neoplasm location**		
Thorax^b^	122	34.3
Gastrointestinal cancer	44	12.4
Head and neck^c^	26	7.3
Genitourinary system	25	7.0
Any/metastases^d^	122	34.3
Other	17	4.8
**Pain type**		
Acute	109	30.6
Chronic	59	16.6
Acute and chronic	29	8.1
Breakthrough	12	3.4
Unknown	147	41.3
**Pain caused by**		
Neoplasm	183	51.4
Surgery	135	37.9
Conservative treatment	38	10.7
**Primary objective**		
Treatment	200	56.2
Supportive care	115	32.3
Prevention	41	11.5
**Intervention type**		
Drug	134	37.6
Procedure	70	19.7
Behavioral	55	15.4
Radiation	5	1.4
Other	92	25.8

a Any involvement of the pharmaceutical industry (either as a sponsor or a collaborator).

b Including breast.

c Excluding central nervous system tumors.

d Trials enrolling patients with any neoplasm type and metastases.

### Assessment of the enrollment criteria

Regarding basic pain characteristics, 59 trials (16.6%) enabled the inclusion of patients with chronic pain and 109 (30.6%) recruited participants with acute pain. In addition, 29 trials (8.1%) allowed for the enrollment of individuals with both acute and chronic pain. In 12 trials (3.4%) patients with breakthrough pain could be included. However, in as many as 156 trials (41.3%) there was no information regarding pain type. We also found that 173 trials (48.6%) recruited patients with pain due to anticancer treatments; these included surgery (*n* = 135; 37.9%) and conservative treatments (*n* = 38; 10.7%).

Ninety-three trials (26.1%) listed as an inclusion criterion the minimal intensity of pain, as determined by numeric rating scale (NRS; median value, 4; IR range, 4–5). We also found that only 9 trials (2.5%) specified the maximal acceptable pain intensity (median value, 3; IR range 3–7). Nine (2.5%) and 1 (0.3%) trials specified the minimal and maximal intensity of pain, respectively, as determined by Brief Pain Inventory (BPI) scale.

Detailed data on the age limits used in clinical trials concerning CRP are shown in Table 2. In most trials (*n* = 343; 96.6%) the minimal age of participants was between 18 and 59 years. Only five trials (1.4%) enrolled solely participants aged 60 or older. We also found that 156 trials (43.8%) excluded participants based on upper age limits. In many trials, upper age limits were within range 66–70, followed by 76–80 years of age (Table 2). The number of trials with the limits of 80 years of age or less (a primary outcome measure) was 126 (35.4%).

**Table 2:** Enrollment criteria in clinical trials in cancer-related pain.

	***n***	**%**
**Lower age limits**		
12–17	8	2.2
18–59	343	96.3
≥60	5	1.4
**Upper age limits**		
≤60	10	2.8
61–65	22	6.2
66–70	38	10.7
71–75	23	6.5
76–80	33	9.3
>80	30	8.4
No limit	200	56.2
**Organs/systems**		
**Psychiatric**		
Strict	46	12.9
Moderate	144	40.4
No restriction	166	46.6
**Renal**		
Strict	22	6.2
Moderate	74	20.8
No restriction	260	73.0
**Hepatic**		
Strict	19	5.3
Moderate	66	18.5
No restriction	271	76.1
**Cardiac**		
Strict	17	4.8
Moderate	74	20.8
No restriction	265	74.4
**Prior or concurrent malignancy**		
Strict	9	2.5
Moderate	27	7.6
No restriction	320	89.9
**Other cardiovascular**		
Strict	7	2.0
Moderate	34	9.6
No restriction	315	88.5
**Pulmonary**		
Strict	5	1.4
Moderate	41	11.5
No restriction	310	87.1
**Bone marrow**		
Strict	3	0.8
Moderate	31	8.7
No restriction	322	90.4
**Broad and imprecise criteria**		
Present	57	16.0
Absent	299	84.0
**Performance score**		
>1 excluded	4	1.1
>2 excluded	38	10.4
>3 excluded	14	3.8
No restriction	300	84.7

We also employed multivariate logistic regression to identify the factors significantly affecting the odds of excluding older adults based on the upper age limits, strict organ-specific criteria, and broad and imprecise criteria. The following factors were included in each logistic regression analysis: primary objective of the trial, neoplasm location, intervention type, phase, sponsor type, center location, number of patients, and whether trial concerned pain following cancer surgery. Detailed results of logistic regression are shown in Supplementary Tables 1–3. On multivariate analysis, higher odds of the limits of 80 years of age or less was found for trials in which the primary objective was the treatment [adjusted odds ratio (aOR), 4.05; confidence interval (CI), 1.86–8.8; *p* < 0.001], trials enrolling patients with certain neoplasm types, especially head and neck cancer (aOR, 4.9; CI, 1.59–15.11; *p* = 0.005) and cancer of the genitourinary system (aOR, 5.52; CI, 1.61–18.92; *p* = 0.006) as well as those conducted in Middle East and North African countries (aOR, 8.64; CI, 1.89–39.56; *p* = 0.005; Supplementary Table 1). On the other hand, the odds of excluding older adults based on the upper age limits was lower in trials in which a drug was used (aOR, 0.24; CI, 0.09–0.64; *p* < 0.01; Supplementary Table 1). However, we found no significant temporal trend toward decreasing the frequency of the upper age limits between 2014 and 2021 (*p* > 0.05).

We also assessed moderate and strict exclusion criteria pertaining to function of different organs and systems. Detailed data on the frequency of different criteria are presented in Table 2. These mostly concerned psychiatry (*n* = 190; 53.4%), specifically different psychiatric disorders (139 trials; 39%), cognitive impairment (83 trials; 23.3%), and substance abuse (65 trials; 18.3%); many trials listed a combination of these criteria. In most of these trials, the exclusion criteria related to psychiatry were fairly broad. Among the trials that listed psychiatric disorders as an exclusion criterion, only 33 (23.7%) provided concrete examples (mostly psychoses and depression) and 39 (28.1%) specified the severity of the disease, while 75 (54%) did not provide either any examples or details about the severity; rather, these referred mostly to “psychiatric diseases,” “psychiatric disorders” or other similar general terms. Among the trials in which cognitive impairment was listed as an exclusion criterion, only 29 (34.9%) provided a specific threshold value of the impairment beyond which the patient was ineligible.

Remarkably, in 46 trials (12.9%) the exclusion criteria pertaining to psychiatric disorders were strict (i.e., a history of a psychiatric disease and/or substance abuse). Other strict exclusion criteria involved impaired renal function (*n* = 22; 6.2%) and impaired hepatic function (*n* = 19; 5.3%). Few trials excluded patients based on the criteria concerning bone marrow function, malignancies, and the pulmonary and cardiovascular systems (Table 2). Overall, strict exclusion criteria (a primary outcome measure) were listed in 95 (26.7%) trials.

Multivariate logistic regression showed that the odds of strict exclusion criteria concerning the function of any organ/system was higher in trials in which a drug was used (aOR, 3.4; CI, 1.42–8.16; *p* = 0.006; Supplementary Table 2). We found no significant temporal trend toward decreasing the frequency of the strict exclusion criteria between 2014 and 2021 (*p* > 0.05).

Apart from the criteria pertaining to function of specific organs and systems, we also assessed broad and imprecise exclusion criteria. These generally did not involve any specific organs or diseases and were open to investigators' interpretation. We found that such criteria were applied in 57 trials (16.0%; Table 2). On multivariate analysis, the odds of broad and imprecise criteria was lower in trials recruiting patients with pain following surgical treatment of cancer (aOR, 0.35; CI, 0.13–0.97; *p* = 0.04) and those conducted in Middle East and North African countries (aOR, 0.08; CI, 0.01–087; *p* = 0.03; Supplementary Table 3). We found no significant trend toward decreasing the frequency of these criteria between 2014 and 2021 (*p* > 0.05).

Moreover, in 59 trials (16.5%) participants were excluded based on low performance status. The scales that were most commonly used included the ECOG scale (*n* = 40; 11.2%), followed by the KS (*n* = 18; 5%). However, only 4 trials (1.1%) excluded participants with the performance score of 2 or more in the ECOG scale or its equivalent in KS (a primary outcome measure; Table 2).

Overall, 241 trials (67.7%) excluded patients based on an upper age limit of 80 years of age or less, or at least one strict or broad and imprecise exclusion criterion, or inadequate performance score. We also noted that among 230 trials without an upper age limit, as many as 118 (51.3%) listed the criteria indirectly increasing the odds of the exclusion of older patients.

We also performed a sub-group analysis for the trials in which a drug was used. This analysis showed that among these trials the most common reason for a patient exclusion were strict organ/system specific criteria (*n* = 54; 40.3%) followed by the upper age limits (*n* = 48; 35.8%), and broad and imprecise criteria (*n* = 27; 20.1%). Very few trials excluded patients with inadequate performance score (*n* = 4; 3%). Overall, 103 trials (76.9%) either explicitly excluded older adults or had high risk of such exclusion.

## Discussion

CRP can occur before the start of anticancer therapy, but it can also be a long-lasting and serious consequence of anticancer treatment including chemotherapy (26), radiotherapy (27), and surgery (28). To cover different causes of pain in cancer patients, we included to our study clinical trials concerning both pain due to the development of the cancer process itself, and pain being a result of chemotherapy, radiotherapy, and surgical treatment of cancer.

The most frequent intervention type in our study were drugs. However, the evidence is mounting that treatment of pain involving solely pharmacotherapy can be ineffective and unsafe (29). Therefore, recently there has been a clear tendency in pain medicine toward comprehensive pain management involving also non-pharmacological options (29, 30). Importantly, as shown by a number of recent systematic reviews and meta-analyses, some non-pharmacological treatments can be effective also in patients with cancer pain (31–35). Therefore, we included to our study clinical trials of non-pharmacological interventions alongside those evaluating the effects of analgesic drugs. Overall, the sample of the included clinical trials reflects the diversity of different causes of CRP and relevant investigational interventions.

Older adults can be excluded from clinical trials in a number of ways. The first and most apparent one involves the use of arbitrary upper age limits. However, older individuals can also be excluded in an indirect way—based on stringent criteria pertaining to function of different organs as well as the performance score (19). An analysis of 495 cancer clinical trials involving 59,300 participants showed that the relaxation of the criteria concerning function of different organs and the performance score would have increased the participation of the elderly by ~50% (22). Moreover, broad and imprecise exclusion criteria are considered to increase risk of excluding older adults (36). Each of these potential barriers was examined in our study.

Overall, we showed that many clinical trials concerning CRP either explicitly exclude older individuals based on the upper age limits or pose high risk of such exclusion due to stringent enrollment criteria. This substantially limits the evidence base for treating older adults with CRP. For instance, more than one third of the analyzed trials had upper age limits. However, the use of arbitrary age limits in clinical trials does not seem to be well-justified in view of the substantial heterogeneity of the aging process. Generally, the chronological age alone does not seem to be a good parameter reflecting an individual's state of health (37, 38). Rather than to exclude patients on the basis of the chronological age, investigators should consider performing geriatric assessment to identify older individuals who may be more susceptible to harms associated with investigational treatments (39, 40).

Remarkably, over a half of the trials that did not have upper age limits, did pose high risk of excluding older patients based on other criteria. This leads to a serious problem—even if older adults are enrolled to clinical trials, they are generally healthier and fitter compared with the average patient encountered in clinical practice. This problem was already reported for other cancer clinical trials (16).

Of note, only 1.4% of the analyzed trials were designed solely for participants aged 60 years or older. For comparison, 5% of the trials concerning the treatment of hematological malignancies enrolled solely participants aged 60 years and older (19). Another study showed that 5% of phase III trials of anticancer treatments published between 2011 and 2014 were dedicated to patients aged 60 years or older (41). Therefore, we believe that investigators should consider the design of more trials enrolling solely the elderly; otherwise, the data on the effects of drugs in these patients can be obtained from subgroup analyses which provide only preliminary evidence of the efficacy and safety of new therapeutic interventions (41).

Regarding the exclusion criteria that may indirectly limit the enrollment of older patients, most of these concerned psychiatry. In fact, 51% of the trials included to this analysis contained such criteria, of which 13.2% were strict. This fairly broad category included psychiatric diseases, substance abuse/addiction, and/or cognitive impairment.

Generally, there are several reasons for which patients with such disorders have been excluded from clinical trials. One of these are problems with obtaining informed consent which is one of the fundamental ethical and legal requirements for a participant's inclusion to a clinical trial. In clinical trials concerning pain an important problem is also a fact that psychiatric disorders may increase risk of abuse of at least some investigational analgesic drugs, especially opioids. The scale of this problem is very serious; in fact it is estimated that three million individuals in the USA and 16 million individuals worldwide have been affected by opioid use disorder—“opioid epidemic” (42). Importantly, psychiatric diseases and other substance abuse are risk factors for opioid misuse and addiction (43). Some other painkillers such as gabapentinoids also have some potential for addiction (44).

Another important reason for frequent exclusion of patients with psychiatric diseases and/or cognitive impairment is associated with the nature of pain which is a subjective symptom whose intensity cannot be assessed by any objective measurement method. Therefore, of primary importance is a clinical trial participant's ability to self-report the effect of an investigational treatment on the pain intensity. However, the ability to self-report pain symptoms is compromised in patients with cognitive impairment (45–47). Furthermore, some psychiatric diseases including schizophrenia and depression can substantially affect pain perception (48, 49).

Thus, on the one hand, the exclusion of patients with psychiatric diseases and/or cognitive impairment has solid justification. However, the prevalence of these disorders in older adults is fairly high. In fact, older age is a risk factor for the development of some neurodegenerative diseases, especially Alzheimer's disease and Parkinson's disease which are associated with serious cognitive impairment (50, 51). It is also known that cognitive impairment is a serious clinical problem in older patients with cancer (52, 53). For instance, one study revealed that its prevalence in individuals with cancer aged 65 years or more at the initiation of the treatment was as high as 46% (54). In patients with hematological malignancies the prevalence of cognitive impairment was even higher—up to 70% (55). Furthermore, some psychiatric diseases are known to occur frequently in older adults. An example of such a disease is depression; a recent systematic review revealed that the global prevalence of major depression in older individuals was 13.3% (56). Importantly, a substantial proportion of patients with depression also has cognitive impairment (57).

Overall, the frequent use of broad exclusion criteria concerning psychiatric disorders and/or cognitive impairment substantially limits the generalizability of the results of clinical trials concerning CRP. In our view, rather than to use broad exclusion criteria (such as “psychiatric disorder,” “mental illness,” or “cognitive impairment”), investigators should consider at least the inclusion of participants with disorders with relatively mild course, for instance mild cognitive impairment. There are several tools that can be used for cognitive assessment including examination of a patient with suspected cognitive impairment. Recent systematic review showed that 14 such tools can be used in clinical research settings, and six of these were evaluated in older patients (58).

A specific group of studies in our sample were trials evaluating the effects of perioperative analgesia in patients undergoing cancer surgery (mostly mastectomy). We consider this group of trials relevant to our study because they are very important for cancer survivors. This results from a fact that acute post-operative pain is a known to be a significant risk factor for developing persistent post-mastectomy pain (PPMP), a syndrome known to negatively affect mood, sleep, cognition, activities of daily living, social interactions, and overall quality of life of breast cancer survivors. Thus, by alleviating acute pain, perioperative analgesia can reduce risk of developing PPMP thereby substantially improving the quality of life of breast cancer survivors (59). This problem is important because PPMP is known to affect up to 50% of women following mastectomy (29). Logistic regression showed that trials concerning the treatment of pain following cancer surgery do not have higher risk of excluding older adults either based on the upper age limits or other criteria.

The main limitation to our study is that we analyzed only trials registered with CT.gov. There are several other registries of clinical trials which make up the World Health Organization (WHO) International Clinical Trials Registry Platform (ICTRP; https://www.who.int/clinical-trials-registry-platform). CT.gov is one of the data providers for the ICTRP. Thus, some clinical trials concerning CRP may have been registered with other registries and are missing from our analysis. However, CT.gov is the most comprehensive register of clinical studies in the world. Of note, a number of studies on the exclusion of older adults from clinical trials have been performed based on trials registered with CT.gov (17–19, 60).

In conclusion, many recent clinical trials concerning CRP either explicitly or implicitly exclude older participants. Given that it is older adults that make up the majority of cancer patients (14), overly restrictive enrollment criteria substantially limit the generalizability of trial results. Sponsors and investigators should consider careful modification of some of the exclusion criteria to improve the enrollment of older participants. In addition, separate trials with less stringent exclusion criteria may be designed to recruit solely older patients.

## Data availability statement

The raw data supporting the conclusions of this article will be made available by the authors, without undue reservation.

## Author contributions

JB and TP contributed to conception and design of the study. KK and EK analyzed clinical trials concerning cancer-related pain. JB wrote the first draft of the manuscript. All authors contributed to manuscript revision, read, and approved the submitted version.

## Funding

This work was supported by funds from the research subvention obtained by the Medical University of Warsaw.

## Conflict of interest

The authors declare that the research was conducted in the absence of any commercial or financial relationships that could be construed as a potential conflict of interest.

## Publisher's note

All claims expressed in this article are solely those of the authors and do not necessarily represent those of their affiliated organizations, or those of the publisher, the editors and the reviewers. Any product that may be evaluated in this article, or claim that may be made by its manufacturer, is not guaranteed or endorsed by the publisher.
