# Supplementary material for: Exclusion of older adults from clinical trials in cancer-related pain
Source: Front Med (Lausanne). 2022 Aug 4;9:945481. doi: 10.3389/fmed.2022.945481 (PMC9385985; doi:10.3389/fmed.2022.945481)
Supplement: Supplementary file 1 [file Data_Sheet_1.docx]

**Supplementary Table 1. Factors affecting the odds of the upper age limits in clinical trials in cancer-related pain.** ^a^including breast; ^b^excluding central nervous system tumors; ^c^trials enrolling patients with any neoplasm type and metastases; ^d^including radiotherapy; ^e^including behavioral interventions; ^f^phase 1/2 and 2/3 trials were excluded from this comparison; ^g^any involvement of the pharmaceutical industry; ^h^Middle East/North Africa; ^i^continuous variable.

| **Adjusted odds Confidence *p* value**  **ratio interval** |
| --- |
| Primary objective  Prevention 2.04 0.68-6.12 0.2  Treatment 4.05 1.86-8.80 <0.001  Supportive care referent - -  Neoplasm location  Chest^a^ 1.62 0.66-3.98 0.29  Gastrointestinal 1.49 0.53-4.22 0.45  Head and neck^b^ 4.90 1.59-15.11 0.005  Genitourinary system 5.52 1.61-18.92 0.006  Any/metastases^c^ referent - -    Intervention type  Drug 0.24 0.09-0.64 0.004  Procedure^d^ 0.45 0.19-1.06 0.07  Other^e^ referent - -  Pain following  cancer surgery  Yes 2.01 0.98-4.15 0.06  No referent - -  Phase^f^  1 2.68 0.31-23.33 0.37  2 1.97 0.57-6.83 0.28  3 3.22 0.98-10.65 0.06  4 1.95 0.60-6.31 0.26  Not applicable referent - -  Sponsor  Non-industry 0.72 0.22-2.33 0.59  Industry^g^ referent - -  Center location  North America 0.35 0.09-1.41 0.14  Europe 0.57 0.14-2.28 0.43  Asia/Far East 4.07 1.01-16.46 0.04  ME/NA^h^ 8.64 1.89-39.56 0.005  Other referent - -  Number of patients^i^ 1.0 1.00-1.00 0.74 |

**Supplementary Table 2.** Factors affecting the odds of strict organ/system-specific exclusion criteria in clinical trials in cancer-related pain. ^a^including breast; ^b^excluding central nervous system tumors; ^c^trials enrolling patients with any neoplasm type and metastases; ^d^including radiotherapy; ^e^including behavioral interventions; ^f^phase 1/2 and 2/3 trials were excluded from this comparison; ^g^any involvement of the pharmaceutical industry; ^h^Middle East/North Africa; ^i^continuous variable.

| **Adjusted odds Confidence *p* value**  **ratio interval** |
| --- |
| Primary objective  Prevention 1.32 0.49-3.56 0.58  Treatment 0.90 0.47-1.76 0.77  Supportive care referent - -  Neoplasm location  Chest^a^ 1.50 0.65-3.50 0.34  Gastrointestinal 0.58 0.18-1.54 0.36  Head and neck^b^ 2.22 0.79-6.19 0.13  Genitourinary system 2.02 0.65-6.23 0.22  Any/metastases^c^ referent - -    Intervention type  Drug 3.40 1.42-8.16 0.006  Procedure^d^ 0.96 0.40-2.28 0.92  Other^e^ referent - -  Pain following  cancer surgery  Yes 1.40 0.68-2.89 0.36  No referent - -  Phase^f^  1 3.87 0.60-25.16 0.16  2 0.73 0.25-2.09 0.55  3 1.56 0.54-4.46 0.41  4 0.35 0.11-1.15 0.08  NA referent - -  Sponsor  Non-industry 1.83 0.71-4.72 0.21  Industry^g^ referent - -  Center location  North America 3.25 0.60-17.62 0.17  Europe 1.94 0.35-10.86 0.45  Asia/Far East 3.80 0.68-21.14 0.13  ME/NA^h^ 3.75 0.61-23.0 0.15  Other referent - -  Number of patients^i^ 1.0 1.00-1.00 0.10 |

**Supplementary Table 3.** Factors affecting the odds of broad and imprecise exclusion criteria in clinical trials in cancer-related pain. ^a^including breast; ^b^excluding central nervous system tumors; ^c^trials enrolling patients with any neoplasm type and metastases; ^d^including radiotherapy; ^e^including behavioral interventions; ^f^phase 1/2 and 2/3 trials were excluded from this comparison; ^g^any involvement of the pharmaceutical industry; ^h^Middle East/North Africa; ^i^continuous variable.

| **Adjusted odds Confidence *p* value**  **ratio interval** |
| --- |
| Primary objective  Prevention 1.54 0.33-7.14 0.58  Treatment 1.28 0.57-2.87 0.54  Supportive care referent - -  Neoplasm location  Chest^a^ 0.66 0.24-1.82 0.42  Gastrointestinal 1.34 0.42-4.31 0.62  Head and neck^b^ 2.23 0.74-6.75 0.16  Genitourinary system 0.90 0.17-4.73 0.90  Any/metastases^c^ referent - -    Intervention type  Drug 0.70 0.22-2.29 0.56  Procedure^d^ 0.77 0.24-2.49 0.66  Other^e^ referent - -  Pain following  cancer surgery  Yes 0.35 0.13-0.97 0.04  No referent - -  Phase^f^  1 0.00 0.00-Inf 0.99  2 2.04 0.53-7.79 0.30  3 3.37 0.92-12.41 0.07  4 1.56 0.36-6.65 0.55  NA referent - -  Sponsor  Non-industry 1.12 0.40-3.17 0.83  Industry^g^ referent - -  Center location  North America 0.35 0.09-1.44 0.15  Europe 0.34 0.08-1.47 0.15  Asia/Far East 0.63 0.15-2.63 0.53  ME/NA 0.08 0.01-0.87 0.03  Other referent - -  Number of patients^h^ 1.0 1.00-1.00 0.32 |
